# Quarantine and testing strategies in contact tracing for SARS-CoV-2: a modelling study

**DOI:** 10.1016/S2468-2667(20)30308-X

**Published:** 2021-01-21

**Authors:** Billy J Quilty, Samuel Clifford, Joel Hellewell, Timothy W Russell, Adam J Kucharski, Stefan Flasche, W John Edmunds, Centre for the Mathematical Modelling of Infectious Diseases COVID-19 working group, Katherine E Atkins, Anna M Foss, Naomi R Waterlow, Kaja Abbas, Rachel Lowe, Carl A B Pearson, Sebastian Funk, Alicia Rosello, Gwenan M Knight, Nikos I Bosse, Simon R Procter, Georgia R Gore-Langton, Alicia Showering, James D Munday, Katharine Sherratt, Thibaut Jombart, Emily S Nightingale, Yang Liu, Christopher I Jarvis, Graham Medley, Oliver Brady, Hamish P Gibbs, David Simons, Jack Williams, Damien C Tully, Stefan Flasche, Sophie R Meakin, Kevin Zandvoort, Fiona Y Sun, Mark Jit, Petra Klepac, Matthew Quaife, Rosalind M Eggo, Frank G Sandmann, Akira Endo, Kiesha Prem, Sam Abbott, Rosanna Barnard, Yung-Wai D Chan, Megan Auzenbergs, Amy Gimma, C Julian Villabona-Arenas, Nicholas G Davies

**Affiliations:** Centre for the Mathematical Modelling of Infectious Diseases, London School of Hygiene & Tropical Medicine, London, UK; a Centre for the Mathematical Modelling of Infectious Diseases, London School of Hygiene & Tropical Medicine, London, UK

## Abstract

**Background:**

In most countries, contacts of confirmed COVID-19 cases are asked to quarantine for 14 days after exposure to limit asymptomatic onward transmission. While theoretically effective, this policy places a substantial social and economic burden on both the individual and wider society, which might result in low adherence and reduced policy effectiveness. We aimed to assess the merit of testing contacts to avert onward transmission and to replace or reduce the length of quarantine for uninfected contacts.

**Methods:**

We used an agent-based model to simulate the viral load dynamics of exposed contacts, and their potential for onward transmission in different quarantine and testing strategies. We compared the performance of quarantines of differing durations, testing with either PCR or lateral flow antigen (LFA) tests at the end of quarantine, and daily LFA testing without quarantine, against the current 14-day quarantine strategy. We also investigated the effect of contact tracing delays and adherence to both quarantine and self-isolation on the effectiveness of each strategy.

**Findings:**

Assuming moderate levels of adherence to quarantine and self-isolation, self-isolation on symptom onset alone can prevent 37% (95% uncertainty interval [UI] 12–56) of onward transmission potential from secondary cases. 14 days of post-exposure quarantine reduces transmission by 59% (95% UI 28–79). Quarantine with release after a negative PCR test 7 days after exposure might avert a similar proportion (54%, 95% UI 31–81; risk ratio [RR] 0·94, 95% UI 0·62–1·24) to that of the 14-day quarantine period, as would quarantine with a negative LFA test 7 days after exposure (50%, 95% UI 28–77; RR 0·88, 0·66–1·11) or daily testing without quarantine for 5 days after tracing (50%, 95% UI 23–81; RR 0·88, 0·60–1·43) if all tests are returned negative. A stronger effect might be possible if individuals isolate more strictly after a positive test and if contacts can be notified faster.

**Interpretation:**

Testing might allow for a substantial reduction in the length of, or replacement of, quarantine with a small excess in transmission risk. Decreasing test and trace delays and increasing adherence will further increase the effectiveness of these strategies. Further research is required to empirically evaluate the potential costs (increased transmission risk, false reassurance) and benefits (reduction in the burden of quarantine, increased adherence) of such strategies before adoption as policy.

**Funding:**

National Institute for Health Research, UK Research and Innovation, Wellcome Trust, EU Horizon 2021, and the Bill & Melinda Gates Foundation.

## Introduction

To break transmission chains of severe acute respiratory syndrome coronavirus 2 (SARS-CoV-2), causative agent of COVID-19, testing of cases and tracing and quarantine of their contacts has been used as a key non-pharmaceutical intervention in many countries. This measure aims to prevent onward transmission from secondary infections (individuals infected by an index case), and has been used successfully to prevent new outbreaks in countries such as South Korea, without the need for lockdown-style measures. As of November, 2020, guidance in the UK was that contact-traced individuals must quarantine from the moment they are traced until 14 days have elapsed from their exposure to the index case. 14 days is the upper bound for the incubation period of the virus,^1^ when more than 95% of eventually symptomatic individuals will have developed symptoms and should subsequently enter a further period of self-isolation (10 days in the UK). However, there is growing evidence that many contacts of cases are unable to effectively quarantine for the entirety of this period, particularly those unable to work from home, or those caring for vulnerable people.^2^ The increasing availability of testing, particularly rapid, low-cost lateral flow antigen (LFA) tests,^3^, ^4^ opens up the possibility of shorter periods of quarantine when combined with a negative test on exit (a test and release strategy), or even the avoidance of quarantine entirely if it is replaced with daily testing. If effective, both these strategies have the potential to substantially reduce the burden of quarantine on uninfected contacts, which could simultaneously improve quarantine adherence and reduce the economic, personal, financial, and social costs of the current policy.

**Research in context**

**Evidence before this study**

During the COVID-19 pandemic, a standard 14-day quarantine period from the day a contact was exposed to an index case has been required in the UK and elsewhere. This approach aims to avert onward transmission during infected contacts' presymptomatic period. This strategy, although a crucial part of the global pandemic response to interrupt transmission chains, places considerable social, financial, and economic pressure on quarantining individuals and society. A search of the literature on Dec 3, 2020, using the terms “quarantine AND test* AND (COVID* OR SARS*) AND effect* AND contact tracing” returned 59 results on PubMed and 1934 results on medRxiv; however, no study had investigated the effect of heterogeneity in viral load or the effectiveness of daily testing without quarantine.

**Added value of this study**

We modelled the individual severe acute respiratory syndrome coronavirus 2 (SARS-CoV-2) viral load trajectories of the contacts of confirmed cases to calculate the effect of a range of quarantine and testing strategies. To the best of our knowledge, this is the first analysis of possible strategies to reduce or replace the quarantine requirement through rapid antigen testing. We found that quarantine until a PCR or lateral flow antigen test on day 7 after exposure (with early release if negative) might avert as much transmission as the 14-day quarantine period. Additionally, daily repeated lateral flow antigen testing of traced contacts for 5 days, with isolation only after a positive test, might allow for the quarantine requirement to be removed with a small increase in transmission risk, which could itself be offset by increased participation and adherence to isolation.

**Implications of all the available evidence**

The ability to identify and isolate infected individuals rapidly and comprehensively is crucial to reduce the incidence of SARS-CoV-2. Testing contacts of confirmed cases might enable the required quarantine periods for uninfected individuals to be substantially shortened, which could dampen the economic and social impact, while potentially increasing compliance. Further research (such as field trials) should be done to evaluate the potential costs (false reassurance, increased transmission risk) and benefits (reduction in quarantine burden, enhanced case detection, increased compliance) of such a policy.

RT-PCR involves amplification and quantification of viral RNA within a nose or throat sample, with a low cycle threshold (Ct) value indicating the presence of greater quantities of viral genetic material and hence a greater likelihood of being infected.^5^ Due to the amplification step, PCR is highly sensitive and specific to the presence of SARS-CoV-2 viral RNA, but requires samples to be sent to a laboratory for processing before return of a result—a process that currently takes an average of 2 days in the UK.^6^ In contrast, LFA tests are pregnancy test-style, point-of-care devices that test for the presence of SARS-CoV-2 antigen and allow for return of results within 15–30 min. LFA tests are reportedly substantially cheaper, and might be produced and distributed more easily and frequently, than PCR tests;^3^ however, the absence of an amplification step results in a lower sensitivity than PCR tests. Despite this decreased sensitivity, the speed at which results are available might allow for repeated testing of individuals, which could enable faster isolation of cases and reduced transmission potential even if the ability to detect infections is lower than with PCR testing.

Testing of traced contacts might detect incubating and asymptomatic cases, allowing for a reduction in the post-exposure quarantine period from 14 days. Key to this is the timing of testing, because testing contacts too early or too late in their infection might lead to false-negative results. Another crucial factor is the delay in testing and tracing—ie, how long has passed since exposure to the index case to the isolation of their contacts—because approximately half of SARS-CoV-2 transmission occurs before the onset of symptoms.^7^ Additionally, the current 14-day quarantine period is poorly adhered to by contacts of cases, with only 10·9% reporting that they did not leave the house in the 14 days after exposure to the index case.^8^ Reducing the length of the quarantine period might increase adherence and therefore avert more transmission overall.

Here, we aimed to evaluate the effect of different quarantine and testing strategies on reducing onward transmission from traced secondary infections using a mathematical model to simulate viral load dynamics, tracing and testing timings, and other relevant parameters. We varied the required post-exposure quarantine period, and the timing, number, and type of tests (standard PCR tests or rapid LFA tests). We also investigated the effect of reducing testing and tracing delays, and the effect of reduced adherence to quarantine. As an alternative to quarantine, we considered daily testing on being traced as a contact, and estimated the number of consecutive daily tests required before leaving isolation that would result in a similar reduction in transmission to that achieved by quarantine.

## Methods

### Contact tracing model of infected individuals

We used a stochastic, individual-based model to simulate an individual's exposure time, viral load trajectory, symptom onset, and tracing and testing timings. The model was specified in such a way as to focus on the cases' infectivity, rather than the number of additional cases generated, and, as such, is independent of the number of secondary or further cases generated.

**Figure 1:**
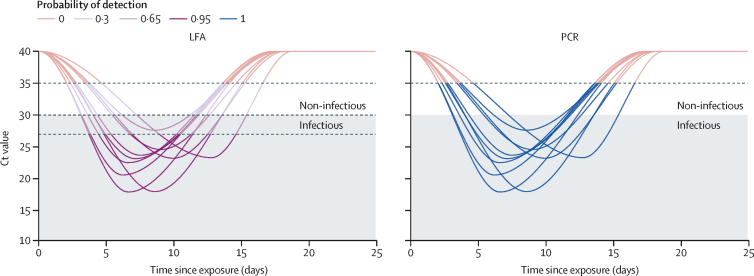
Simulated Ct curves for ten individuals infected with SARS-CoV-2 Dashed lines represent thresholds for detection probabilities^9^ and the shaded region, with boundary at Ct 30, indicates the time during which individuals are considered infectious. One of the individuals never reaches Ct 30 and hence they are considered to not be infectious; however, they will be detectable by PCR and with probability 0·3 for LFA during *t*ε(5–13). Ct=cycle threshold. SARS-CoV-2=severe acute respiratory syndrome coronavirus disease 2. LFA=lateral flow antigen.

For each individual in the model (index cases and secondary cases), we simulated a viral load trajectory of Ct values over the course of infection (figure 1) using published data to inform our choice of parameters. Each curve is parameterised by a baseline Ct level, a peak Ct value, and an end time, representing a return to baseline. We assumed a baseline Ct of 40 on exposure (ie, negative for SARS-CoV-2). The timing of the peak Ct was sampled from the incubation period (time from exposure to onset of symptoms) using the pooled log-normal distribution from a published meta-analysis.^10^ The peak Ct value is normally distributed with mean 22·3 and SD of 4·2^9^ and the time of cessation of viral shedding, a return to baseline, is parameterised as normally distributed with mean 17 days after exposure and SD of 0·94 days for symptomatic individuals,^11^ with asymptomatic individuals having a duration that is 40% shorter.^10^ The peak and end times are drawn, for each individual, in such a way that each individual is at the same quantile, q, in the cumulative densities of each distribution; this guarantees that the ordering of peak and end is maintained and that there are no rapid returns to baseline Ct after a slow transition to peak Ct. We then fit a cubic Hermite spline^12^ to the generated exposure, peak, and end values for each individual, constraining the slope of the curve to be zero at each of them, to simulate viral load kinetics (in Ct) over the course of infection. We assumed that an individual is infectious during the time period that their Ct value is less than 30.^13^ If an individual's Ct trajectory does not drop below 30, they are considered to never be infectious and therefore not relevant for transmission. We assumed individuals are uniformly infectious during this period of Ct less than 30.

We simulated index cases as individuals who become exposed, then infectious, at which point they begin exposing their contacts and generating secondary cases. Once the index cases develop symptoms, they begin a period of self-isolation when they are unable to generate additional secondary cases. We assumed that 1 day after symptom onset, they seek out and have a PCR test that is returned positive, which begins the process of contact tracing. Based on the latest National Health Service test and trace data, we assumed that it takes a delay of 3 days from the sample being taken to contacts being instructed to quarantine.^14^ To investigate the effect of faster contact tracing (eg, through rapid testing and application-based tracing^15^), we considered halved delays (1·5 days) and instant test and trace (0 days) as a sensitivity analysis.

### Quarantine and testing strategies

We assumed that all contacts are successfully identified and traced and, that once traced, are subject to one of several strategies designed to avert onward transmission. In the quarantine-based strategy, we investigated quarantine durations of 0 days, 3 days, 5 days, 7 days, 10 days, and 14 days post exposure to the index case, with either no testing or testing with PCR or LFA tests on the final day of the specified quarantine period (to highlight the effect of said test at the end of quarantine). However, if the end-of-quarantine test is scheduled to occur before the time of the secondary case's tracing, we assumed that they are tested as soon as they are traced; hence, a 0-day quarantine with a test will be equivalent to an immediate test and release strategy. In the daily testing strategy, contacts are required to take an LFA test every day for 1 day, 3 days, 5 days, 7 days, 10 days, or 14 days after they are traced and are not required to quarantine unless they either develop symptoms or test positive. Secondary cases displaying symptoms at any point post exposure, or testing positive at any time, will then isolate until 10 days have passed since onset of symptoms.^16^ Given that asymptomatic secondary cases never develop symptoms, they will self-isolate only if they test positive. We sampled the proportion of secondary infections that are asymptomatic from a beta distribution, which has a median of 31% (95% CI 24–38;^17^ table). Further details on the model parameters are provided in the table.

**Table:** Model parameters and their values in simulation of cases' infection histories and testing

	**Description**	**Value**	**Source**
Incubation period	Time from exposure to onset of symptoms	Log-normal (log-mean 1·63, log-SD 0·5), median 5·1 days, IQR 3·9–6·7 days, 95% CI 2·3–11·5 days	McAloon and colleagues^10^
Infectious period	Time for which Ct is less than 30	Symptomatic individuals mean 7·56 days, SD 1·54 days; asymptomatic individuals mean 4·32 days, SD 1·09 days	Derived
Asymptomatic fraction of secondary cases, *a*	Proportion of infections that are asymptomatic	Beta (alpha 51, beta 115), median 0·31, IQR 0·28–0·33, 95% CI 0·24–0·38	Derived from quantile matching 95% prediction interval^17^

Ct=cycle threshold.

The probability of detecting an infected and possibly infectious individual depends on their Ct value at the time of testing, and is drawn from their individual Ct trajectory (figure 1). For PCR, we assumed that the probability of detection is 100% for Ct below 35 and 0% above 35. For LFA, we approximated the probability of detection is 95% for Ct below 27, 65% for Ct from 27 to 30, 30% for Ct from 30 to 35, and 0% above 35, approximated based on the results of the Innova rapid antigen test evaluation.^4^ As a sensitivity analysis to investigate the effect of lower Innova LFA sensitivity, we used the probability of detection for a given Ct as reported in the Liverpool Mass Testing Pilot.^18^

As a moderate baseline scenario, we assumed that 50% of individuals adhere to quarantine and 67% adhere to self-isolation guidelines. To investigate the effect of increased or reduced adherence to quarantine and self-isolation on the effectiveness of the programme, we considered adherences of 100% and 0% for post-tracing quarantine, and 100% and 0% for self-isolation after a positive test or symptom onset. We assumed adherence as a binary variable (adhering or not-adhering) for each individual by sampling from a Bernoulli distribution with the probability given by the proportion adhering.

### Transmission potential

For each secondary case, we considered the infectious period as the period of time when the individual's Ct values are less than 30. We then calculated the amount of the infectious period spent in quarantine, or in self-isolation due to onset of symptoms or after a positive test, as transmission potential averted. Assuming that the majority of SARS-CoV-2 transmission is driven by superspreading events,^19^ we report the uncertainty associated with the average secondary transmission potential averted per superspreading event by simulating 1000 index cases with ten secondary cases. We calculated the median and inner 50% and 95% ranges for the sum of the secondary cases' infectious periods spent in quarantine or self-isolation divided by the sum of secondary cases' infectious periods if there were no quarantine or self-isolation requirements. Because the model considers averting this transmission rather than focusing on the generation of additional cases, the average amount of infectivity in secondary cases averted by quarantine or testing, or both, is independent of the number of additional cases generated, and the choice of the number of secondary cases affects the width of the uncertainty intervals (UIs; here we consider a reasonable upper bound on secondary cases based on superspreading, as mentioned, in an attempt to faithfully characterise real-world uncertainty). We also calculated the risk ratio (RR) of transmission averted by the given strategy compared with the baseline scenario (a 14-day quarantine period with no testing, 3 days from testing of the index case to tracing, 50% adherence to quarantine, and 67% adherence to self-isolation).

In our calculation of the transmission potential averted, we considered that in the case that no transmission is averted, an individual will be as infectious as if there were no testing or quarantine. In such a case, that individual is likely to go on to infect a number of additional individuals, *R*, which is distributed with mean *R*_0_ and dispersion *k*. With a fraction, *a*, of their infectivity prevented, an infectious individual is expected to infect *(1–a)R* individuals. Hence, the transmission potential averted can be thought of as a linear scaling of *R*.

The model was coded in R, version 4.0.3, and the entire code required to reproduce this analysis is available online.

### Role of the funding source

The funders of this study had no role in study design, data collection, data analysis, data interpretation, or writing of the report. All authors had full access to all of the data and the final responsibility to submit for publication.

## Results

**Figure 2:**
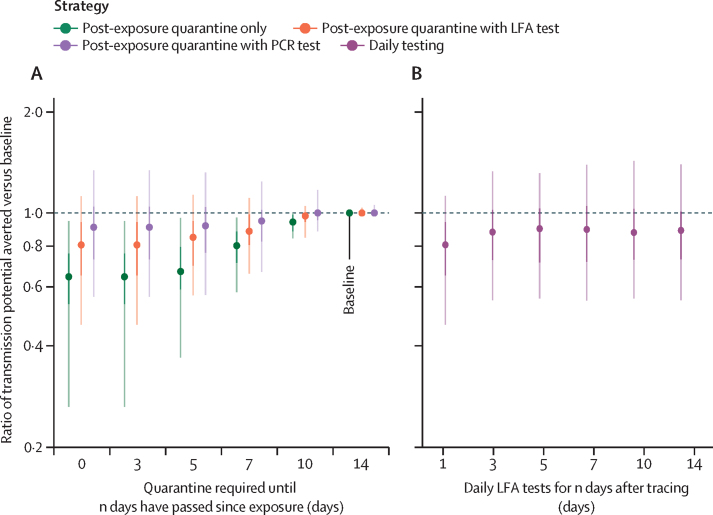
Transmission potential averted with quarantine-based strategies and daily testing strategies Ratio was calculated as the sum of days of secondary cases' infectious periods spent in quarantine or self-isolation divided by the sum of days of secondary cases' infectious periods. Ratios are shown for each strategy versus the baseline of 14 days' quarantine with no testing, for quarantine-based strategies (quarantine required from time of tracing until n days have passed since exposure, either with or without a test on the final day; A) and daily testing strategies (daily LFA tests without quarantine for n days from tracing, isolating only after a positive test result; B). Quarantine and self-isolation adherence were assumed to be 50% and 67%, respectively. The delay from an index case's positive test until the tracing of secondary cases was assumed to be 3 days (current average).^16^ Central bars indicate the median ratio for a given strategy, with 95% and 50% uncertainty intervals indicated by light and dark shaded bars, respectively. LFA=lateral flow antigen.

According to our model, relying only on 67% of eventually symptomatic people self-isolating on developing symptoms, 37% (95% UI 12–56) of transmission might be averted from secondary infections, with an RR of 0·68 (95% UI 0·22–0·95) compared with the baseline scenario. By tracing contacts and instructing them to self-isolate for a period of time after their last exposure to the index case, additional transmission might be averted from asymptomatic and presymptomatic secondary cases (figure 2; appendix p 1). The amount of transmission averted rises to 46% (95% UI 16–63) with an RR of 0·81 (95% UI 0·49–0·99) at 7 days post exposure; to 54% (95% UI 24–71) with an RR of 0·92 (95% UI 0·72–1·00) at 10 days post exposure; and to 59% (95% UI 28–79, baseline) at 14 days post exposure.

The amount of transmission potential averted can be increased if LFA or PCR testing is done on the final day of quarantine (or on tracing, if the specified quarantine period ends before a case is traced) and people who receive a negative result are released. The introduction of an immediate test is estimated to avert 49% (95% UI 24–68) of transmission with an LFA test (RR 0·79, 95% UI 0·38–1·32) and 53% (95% UI 24–79) of transmission with a PCR test (RR 0·89, 95% UI 0·62–1·51; figure 2; appendix p 1). However, the greater time spent in quarantine waiting for a PCR test result might avert additional transmission, although these delays might not be desirable features of a test and trace system. Shorter quarantines with a test on the final day might avert a similar amount of transmission to that of the current 14-day quarantine without a test—ie, 7 days with an LFA test (50%, 95% UI 28–77; RR 0·88, 95% UI 0·66–1·11), 10 days with an LFA test (56%, 95% UI 32–81; RR 0·95, 95% UI 0·80–1·02), 7 days with a PCR test (54%, 95% UI 31–81; RR 0·94, 95% UI 0·62–1·24), and 10 days with a PCR test (56%, 95% UI 33–81; RR 1·00, 95% UI 0·84–1·09). As the quarantine period increases in length, the relative contribution of a test is lessened, as the majority of the infectious period is spent in quarantine. With 14 days of mandatory quarantine, 59% (95% UI 28–79, baseline) of transmission is averted with no testing, and 59% (95% UI 33–82) of transmission is averted (RR 1·00, 95% UI 1·00–1·04) with either a PCR or LFA test (figure 2). Although no shorter quarantine strategy with testing might exceed the median amount averted by the 14-day quarantine, all PCR testing strategies evaluated avert equivalent amounts of transmission within the 50% uncertainty bounds.

If traced contacts are required to take a daily LFA test for n days after tracing instead of entering quarantine, 5 days of testing might avert 50% (95% UI 23–81; RR 0·88, 95% UI 0·60–1·43) of transmission, with additional days of testing averting a similar amount (figure 2).

**Figure 3:**
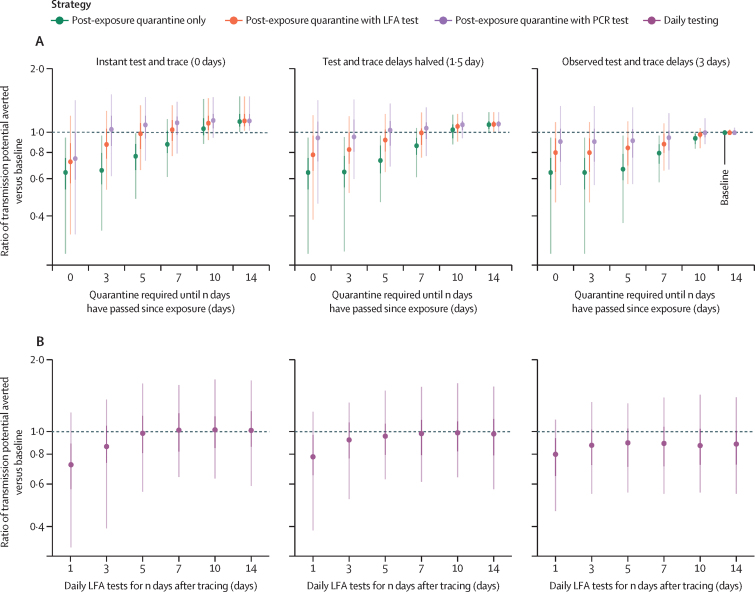
Transmission potential averted with reduced test and trace delays Ratio was calculated as the sum of days of secondary cases' infectious periods spent in quarantine or self-isolation divided by the sum of days of secondary cases' infectious periods. Ratios are shown for each strategy versus the baseline of 14 days' quarantine with no testing, for quarantine-based strategies (quarantine required from time of tracing until n days have passed since exposure, either with or without a test on the final day; A) and daily testing strategies (daily LFA tests without quarantine for n days from tracing, isolating only after a positive test result; B). Quarantine and self-isolation adherence were assumed to be 50% and 67%, respectively. The delay from an index case's positive test until the tracing of secondary cases was assumed to be 3 days (current average^16^) in the baseline scenario, with halved and eliminated delays investigated. Central bars indicate the median ratio for a given strategy, with 95% and 50% uncertainty intervals indicated by light and dark shaded bars, respectively. LFA=lateral flow antigen.

Our model suggest that if test and trace delays (ie, the time from the index case having a test to the tracing of their contacts) can be reduced, shorter quarantines might become more viable, because the proportion of the infectious period spent in the community before tracing decreases (figure 3; appendix p 2). For example, if test and trace delays can be reduced to zero (ie, through digital contact tracing), the median RR of a 10-day quarantine with no testing might exceed the effect of the 14-day quarantine with a 3-day test and trace delay (RR 1·07, 95% UI 0·80–1·64). The effect of daily testing strategies might also exceed the effect of the current 14-day strategy with zero delays (5 days of LFA testing RR 1·01, 95% UI 0·54–1·62); however, because secondary infections will be traced earlier in their infection when viral loads are lower, the likelihood of false negatives increases, and additional days of testing (ie, 7–10 days) might be required (figure 3; appendix p 2).

**Figure 4:**
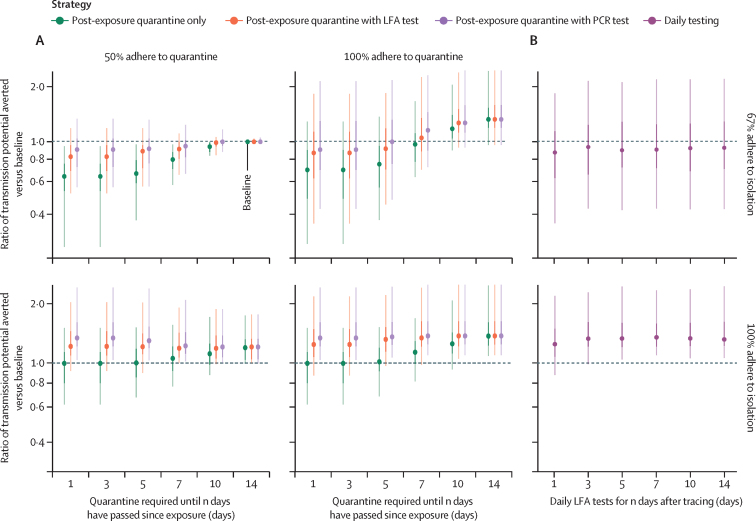
Transmission potential averted with increased adherence to self-isolation and quarantine Ratio was calculated as the sum of days of secondary cases' infectious periods spent in quarantine or self-isolation divided by the sum of days of secondary cases' infectious periods. Ratios are shown for each strategy versus the baseline of 14 days' quarantine with no testing, for quarantine-based strategies (quarantine required from time of tracing until n days have passed since exposure, either with or without a test on the final day; A) and daily testing strategies (daily LFA tests without quarantine for n days from tracing, isolating only after a positive test result; B). Quarantine and self-isolation adherence were assumed to be 50% and 67%, respectively, in the baseline scenario, with 100% explored for both. The delay from an index case's positive test until the tracing of secondary cases was assumed to be 3 days (current average).^16^ Central bars indicate the median ratio for a given strategy, with 95% and 50% uncertainty intervals indicated by light and dark shaded bars, respectively. LFA=lateral flow antigen.

We found that if rates of adherence to quarantine and self-isolation can be boosted, substantial increases in effect over that of the baseline 14-day quarantine policy might be achieved, assuming that in the baseline scenario, 50% of individuals adhere to quarantine and 67% of individuals adhere to post-symptom or post-positive test self-isolation (figure 4; appendix p 3). For example, if individuals adhere perfectly to self-isolation after a positive test in a daily testing scenario, 5 days of testing with LFA after tracing might avert 80% (95% UI 66–89) of transmission (RR 1·33, 95% UI 1·04–2·42).

If more conservative estimates of LFA sensitivity are used,^18^ lateral flow tests might be less efficacious, with quarantine and a negative LFA test at 7 days after exposure averting 44% (95% UI 17–66) of transmission (RR 0·79, 95% UI 0·54–1·04) and 5 days of LFA testing without quarantine averting 43% (95% UI 17–64) of transmission (RR 0·77, 95% UI 0·34– 1·16; appendix p 4).

## Discussion

Using a model combining SARS-CoV-2 viral load dynamics with a range of possible quarantine and testing strategies for contact tracing, we estimate that the recommended 14 days of quarantine after last exposure from a confirmed case can prevent 59% (95% UI 28–79) of onward transmission from secondary cases, assuming 50% adherence to quarantine and a total delay of 3 days from the index case having a test to the tracing of their contacts. Assuming the same level of adherence for quarantine and 67% adherence to self-isolation after symptom onset or a positive test, an LFA test 7 days after exposure with quarantine from tracing until testing or alternatively daily testing with LFA tests for 5 days after tracing might avert a similar proportion to that of the 14-day quarantine (RR 0·88, 95% UI 0·66–1·11 and RR 0·88, 95% UI 0·60–1·43, respectively), if all tests are negative, potentially allowing for the reduction of or removal of the quarantine requirement for traced contacts. In strategies requiring quarantine, the additional benefit of testing diminishes with longer quarantine durations, because infectious people spend a greater proportion of their infectious period in quarantine and have a higher probability of developing symptoms (if ever symptomatic) and self-isolating. PCR testing performs better than LFA testing (by averting a greater amount of transmission); however, PCR testing might be limited by the requirement to process samples in a laboratory, a process which has inherent delays (24 h minimum) and logistical limitations (transporting of samples, requirement for skilled staff).

We found that the effectiveness of contact tracing can be limited by low adherence to quarantine and isolation. Data on adherence rates are sparse. A UK survey found that only 10·9% of contacts adhere to quarantine and 18·2% adhere to self-isolation;^8^ however, adherence was defined as not leaving the house at all in the 14 days, with most breaches being brief and of low transmission risk—eg, solo outdoor exercise. Hence, we assumed a higher, moderate baseline of 50% of individuals fully adhering to quarantine (and therefore having their transmission potential reduced to zero), which we assumed increased to 67% for self-isolation after symptom onset or a positive test, which might better reflect the rate of public involvement in contact tracing. It is possible that some of the factors inhibiting adherence to the current 14-day quarantine are difficulty in completing fully due to social and financial burdens, and low perception of the risk to others given an unknown case status.^20^ As such, reducing the duration of quarantine and increasing the use of tests to compensate might raise adherence by making it easier to complete a full term, and by making cases aware that they might be infectious. Investigating this assumption in our modelling, we found that raised adherence increases the benefit of both short quarantines with testing (at the end of quarantine) and daily testing, beyond that of the current 14-day quarantine. As well as the boost in adherence, which might arise through these strategies, effort should be made to increase adherence through other methods, such as increasing trust in government and public health advice; producing clear guidance on the specified contact tracing protocol; increasing the perceived importance of quarantine in reducing transmission; building strong local and social support networks; and increasing the level of income support and provision of other supplies.^20^ Further work on COVID-19 quarantine adherence is required to understand how quarantined individuals behave and whether isolation of cases and suspected cases in hotels or hospitals might be considered to prevent onward transmission.

The ability of any contact tracing programme to minimise the transmission potential of secondary cases is limited substantially by delays from the testing of index cases to the tracing of their contacts, because secondary cases might have been transmitting for a number of days in the community during the time that contact tracing is taking place. If these delays can be reduced through the adoption of rapid testing, rapid digital contact tracing,^15^ or both, a greater overall proportion of transmission might be averted; eg, 68% (95% UI 38–86) of transmission might be averted from secondary cases if contacts can be notified as soon as a case is tested (assuming the same baseline assumptions for adherence). As such, great emphasis should be put on monitoring and reducing the time taken to reach secondary cases. However, if such reductions are achieved, a proportionally longer quarantine period or greater number of days of testing will be required to ensure that quarantine or testing overlaps with the period when contacts are most infectious.

Our study has several limitations. In this analysis, we have focused on the potential for quarantine and testing to reduce the transmission potential of traced secondary infections and have not evaluated the number, and cost, of tests that might be required, nor the possibility of false positives, which—despite the high specificity of PCR and LFA—might arise in mass testing of asymptomatic individuals. However, in the context of contact tracing, where prevalence of SARS-CoV-2 among contacts of confirmed cases is likely to be higher than among the general public, this is unlikely to lead to a low positive predictive value. Due to a lack of currently available data, we have assumed that index cases seek out and take a PCR test 1 day after the onset of symptoms. We do not consider other aspects of the test and trace system that might result in poor outcomes, such as the fraction of index cases that do not engage with the service,^21^ variation in the number of cases generated by each index case,^22^ or the proportion of secondary cases missed by tracers.^23^ Additionally, we do not consider the quarantine, or testing of the contacts of contacts (ie, household members) who test positive, or both, which might constitute a substantial additional effect. For our assumptions of adherence to quarantine and self-isolation, we selected static, moderate values of the proportion of contacts who adhere to each. It is probable that adherence varies (eg, between individuals and waning with the duration of quarantine); however, in the absence of suitable data on the functional form of such changes in adherence, we take a parsimonious approach to modelling adherence.

One of the simplifying assumptions we have made is that the Ct curve is a reasonable proxy for both probability of detection by testing (with both PCR and LFA) and potential for transmission. Alternative parameterisations of transmission potential are possible,^24^ but unresolved challenges in comparing testing approaches with the transmission potential based on a combination of an incubation period^9^ and infectivity relative to onset of symptoms^25^ include the need to convert from PCR sensitivity curves^26^, ^27^ to LFA in such a way that the timing and height of the two curves are matched meaningfully. A more complete picture of daily testing would require mapping a curve of viral load to one of test sensitivity and one of infectivity. Additionally, while we model viral load and the sensitivity of LFA relative to Ct by PCR in line with the University of Oxford and Public Health England evaluation,^4^ Ct values might not be directly comparable between laboratories if different RT-PCR platforms are used.^5^ As such, we have provided a sensitivity analysis using the lower reported sensitivities of LFA in the Liverpool Mass Testing Pilot^18^ and discussion of results in the context of other studies (appendix p 4).

We have shown that quarantine with a test on day 7 post exposure or 5 days of LFA tests could reduce the transmission potential from secondary cases notified through contact tracing to similar levels to that of a 14-day quarantine without testing. However, factoring in structural issues in contact tracing, such as testing and tracing delays and poor adherence of traced cases, greatly reduces the ability of quarantine and testing to reduce onward transmission, and addressing these should be a focus of policy.

## Data sharing

The entire code and data required to reproduce this analysis are available online.
