# Correction to *Lancet Public Health* 2021; 6: e175–83

**DOI:** 10.1016/S2468-2667(21)00111-0

**Published:** 2021-05-19

*Quilty B J, Clifford S, Hellewell J, et al. Quarantine and testing strategies in contact tracing for SARS-CoV-2: a modelling study.* Lancet Public Health *2021;* 6: *e175–83*—In this Article, the R code used for the analyses has been corrected. As a result, data throughout the text and in figures 2, 3, and 4 have been corrected. Some data in the appendix have also been corrected. The consequence of this error was to underestimate the effectiveness of strategies that involve testing in comparison to quarantine alone. Strategies involving PCR testing combined with quarantine, testing with a lateral flow antigen test 7 days after quarantine, or several days of testing with lateral flow antigen tests are now estimated to more closely correspond to or exceed the effectiveness of a 14-day quarantine period without testing. These corrections have been made as of May 18, 2021.

