# Supplementary material for: Quarantine and testing strategies in contact tracing for SARS-CoV-2: a modelling study
Source: Lancet Public Health. 2021 Jan 21;6(3):e175–83. doi: 10.1016/S2468-2667(20)30308-X (PMC7826085; doi:10.1016/S2468-2667(20)30308-X)
Supplement: Supplementary appendix [file mmc1.pdf]

THE LANCET
Public Health

Supplementary appendix

This appendix formed part of the original submission and has been peer reviewed. We post it as supplied by the authors.

Supplement to: Quilty BJ, Clifford S, Hellewell J, et al. Quarantine and testing strategies in contact tracing for SARS-CoV-2: a modelling study. *Lancet Public Health* 2021; published online Jan 20. https://doi.org/10.1016/S2468-2667(20)30308-X.

Supplementary appendix

Figure S1: Transmission potential averted **1**

Figure S2: Transmission potential averted with reduced test and trace delays **2**

Figure S3: Transmission potential averted with reduced or increased adherence **3**

Figure S4: Ratio of transmission potential averted with values of sensitivity reported in the Liverpool mass asymptomatic testing trial **4**

Lateral flow antigen (LFA) sensitivity **4**

Group authorship **4**

Supplementary references **5**

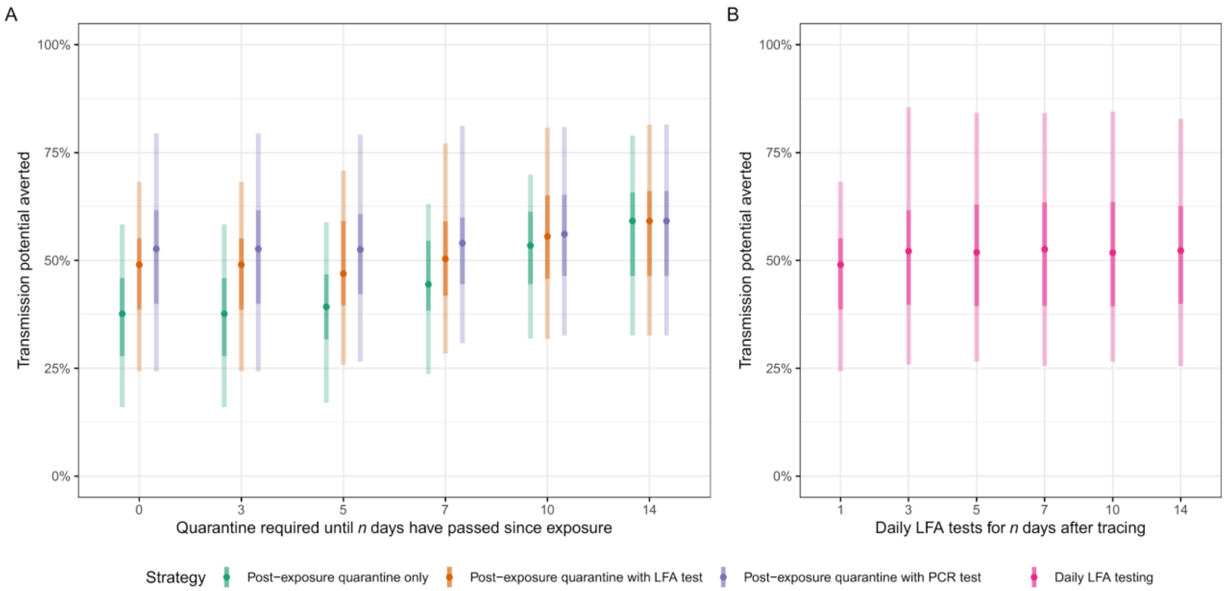

Figure S1: **Transmission potential averted** (sum of days of secondary cases' infectious periods spent in quarantine or self-isolation/ sum of days of secondary cases' infectious periods) for each strategy with quarantine-based strategies (quarantine required from time of tracing until *n* days have passed since exposure, either with or without a test on the final day) in **A** and daily testing strategies (daily lateral-flow antigen tests without quarantine for *n* days from tracing, isolating only upon a positive test result) in **B**. Quarantine and self-isolation adherence assumed to be 50% and 67%, respectively. The delay from index case's positive test until the tracing of secondary cases is assumed to be 3 days (current average). Central bars indicate the median ratio for a given strategy, with 95% and 50% uncertainty intervals indicated by light and dark shaded bars, respectively.

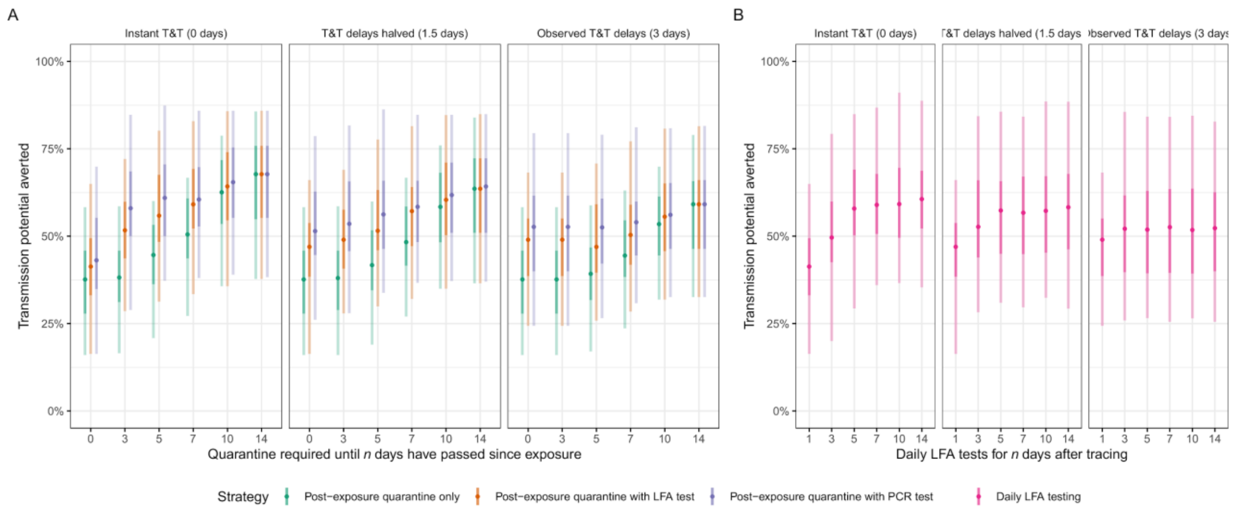

Figure S2: **Transmission potential averted with reduced test and trace delays** (sum of days of secondary cases' infectious periods spent in quarantine or self-isolation/ sum of days of secondary cases' infectious periods) for each strategy with quarantine-based strategies (quarantine required from time of tracing until *n* days have passed since exposure, either with or without a test on the final day) in **A** and daily testing strategies (daily lateral-flow antigen tests without quarantine for *n* days from tracing, isolating only upon a positive test result) in **B**. Quarantine and self-isolation adherence assumed to be 50% and 67%, respectively. The delay from index case's positive test until the tracing of secondary cases is assumed to be 3 days (current average), with sensitivity analysis with halved delays or instant Test & Trace. Central bars indicate the median ratio for a given strategy, with 95% and 50% uncertainty intervals indicated by light and dark shaded bars, respectively.

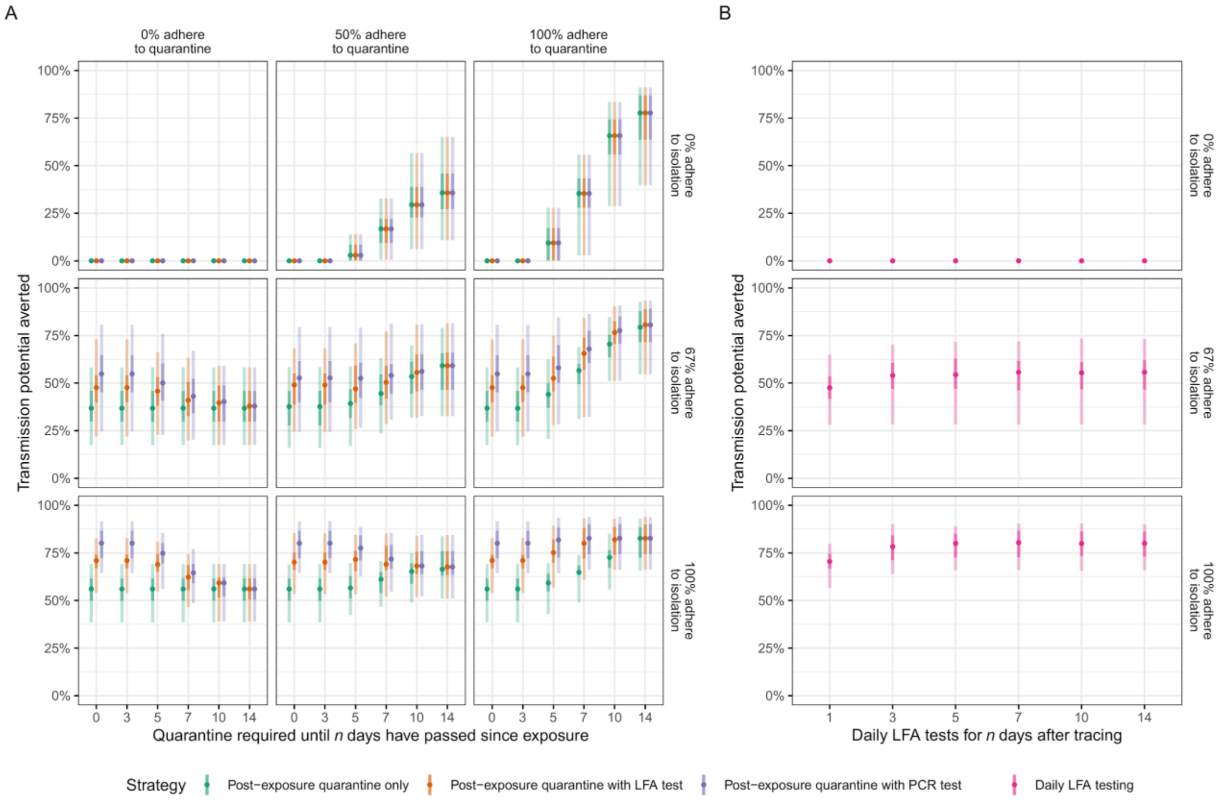

Figure S3: **Transmission potential averted with reduced or increased adherence** (sum of days of secondary cases' infectious periods spent in quarantine or self-isolation/ sum of days of secondary cases' infectious periods) for each strategy with quarantine-based strategies (quarantine required from time of tracing until *n* days have passed since exposure, either with or without a test on the final day) in **A** and daily testing strategies (daily lateral-flow antigen tests without quarantine for *n* days from tracing, isolating only upon a positive test result) in **B**. Quarantine and self-isolation adherence assumed to be 50% and 67%, respectively in the base case, with sensitivity analysis values of 0% and 100% for each. The delay from index case's positive test until the tracing of secondary cases is assumed to be 3 days (current average). Central bars indicate the median ratio for a given strategy, with 95% and 50% uncertainty intervals indicated by light and dark shaded bars, respectively.

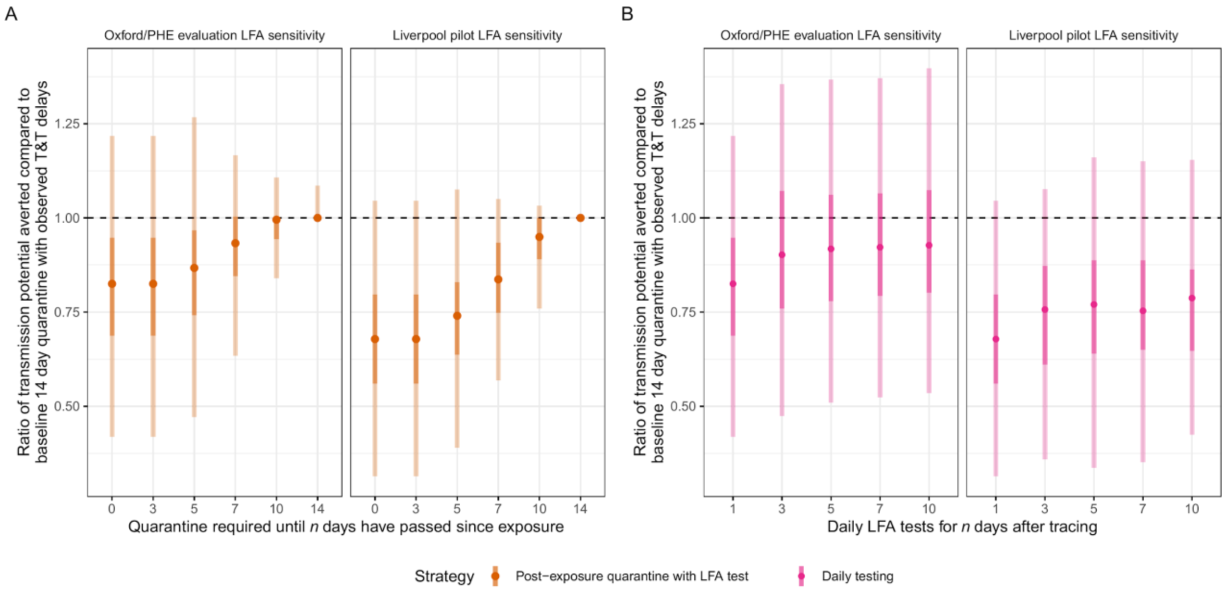

Figure S4: **Ratio of transmission potential averted with values of sensitivity reported in the Liverpool mass asymptomatic testing trial** (sum of days of secondary cases' infectious periods spent in quarantine or self-isolation/ sum of days of secondary cases' infectious periods) for each strategy vs the baseline of 14 days quarantine with no testing, with quarantine-based strategies (quarantine required from time of tracing until *n* days have passed since exposure, either with or without a test on the final day) in **A** and daily testing strategies (daily lateral-flow antigen tests without quarantine for *n* days from tracing, isolating only upon a positive test result) in **B**. Quarantine and self-isolation adherence assumed to be 50% and 67%, respectively, in the baseline scenario. The delay from index case's positive test until the tracing of secondary cases is assumed to be 3 days (current average). Central bars indicate the median ratio for a given strategy, with 95% and 50% uncertainty intervals indicated by light and dark shaded bars, respectively.

Lateral flow antigen (LFA) sensitivity

As a sensitivity analysis for the performance of lateral-flow antigen testing, we use the interim reported results of the Liverpool Community Testing pilot which used the Innova lateral flow antigen test to test asymptomatic individuals in real-world, self-administered use. The mean sensitivities reported by Ct band were 5.3% for 30-35, 8.3% for 25-30, 54.5% for 20-25, and 82.4% for <20, lower than that reported by University of Oxford/Public Health England in their evaluation. We find that this results in a lower effect estimate (Figure S4), however, the uncertainty intervals for daily testing continue to cross the null when compared to the 14-day quarantine period. It should be noted that the difference in apparent sensitivity may be influenced by variation in the PCR assay used and therefore cycle threshold measured by different labs; in the Liverpool trial, the median Ct in asymptomatics was 22.1[1] whereas multiple studies report central estimates of Ct in asymptomatic individuals as being in the range 27-35[2–8]. This 5-10 Ct shift may explain the lower sensitivity of lateral flow tests reported in Liverpool. In order to address this uncertainty, protocols should be clearly reported to enable standardisation and comparison of the viral load of individuals, preferably in SI units. Alternatively, a component of the lower sensitivity observed in the Liverpool Pilot may be attributable to inferior sampling practice by untrained members of the public in real-world conditions.

Group authorship

The following authors were part of the Centre for Mathematical Modelling of Infectious Disease COVID-19 Working Group: Alicia Rosello, Kevin van Zandvoort, Yung-Wai Desmond Chan, Rachel Lowe, Jack Williams, Hamish P Gibbs, James D Munday, Naomi R Waterlow, Rosalind M Eggo, Gwenan M Knight, Amy Gimma, Simon R Procter, Damien C Tully, Emily S Nightingale, Petra Klepac, Kiesha Prem, Fiona Yueqian Sun, Kaja Abbas, Alicia Showering, Akira Endo, Sebastian Funk, Anna M Foss, Thibaut Jombart, Sam Abbott, Rosanna C Barnard, Yang Liu, Carl A B Pearson, Katharine Sherratt, Christopher I Jarvis, Matthew Quaife, Katherine E. Atkins, Oliver Brady, C Julian

Villabona-Arenas, Sophie R Meakin, Graham Medley, Frank G Sandmann, David Simons, Mark Jit, Nicholas G. Davies, Nikos I Bosse.

Affiliation: Centre for Mathematical Modelling of Infectious Diseases, London School of Hygiene and Tropical Medicine, London WC1E 7HT, United Kingdom

The following funding sources are acknowledged as providing funding for the working group authors. BBSRC LIDP (BB/M009513/1: DS). This research was partly funded by the Bill & Melinda Gates Foundation (INV-001754: MQ; INV-003174: KP, MJ, YL; NTD Modelling Consortium OPP1184344: CABP, GFM; OPP1180644: SRP; OPP1183986: ESN). BMGF (OPP1157270: KA). DFID/Wellcome Trust (Epidemic Preparedness Coronavirus research programme 221303/Z/20/Z: CABP, KvZ). EDCTP2 (RIA2020EF-2983-CSIGN: HPG). Elrha R2HC/UK DFID/Wellcome Trust/This research was partly funded by the National Institute for Health Research (NIHR) using UK aid from the UK Government to support global health research. The views expressed in this publication are those of the author(s) and not necessarily those of the NIHR or the UK Department of Health and Social Care (KvZ). ERC Starting Grant (#757699: MQ). This project has received funding from the European Union's Horizon 2020 research and innovation programme - project EpiPose (101003688: KP, MJ, PK, RCB, YL). This research was partly funded by the Global Challenges Research Fund (GCRF) project 'RECAP' managed through RCUK and ESRC (ES/P010873/1: AG, CIJ, TJ). HDR UK (MR/S003975/1: RME). MRC (MR/N013638/1: NRW). Nakajima Foundation (AE). NIHR (16/137/109: FYS, MJ, YL; Health Protection Research Unit for Immunisation NIHR200929: NGD; Health Protection Research Unit for Modelling Methodology HPRU-2012-10096: TJ; NIHR200908: RME; NIHR200929: FGS, MJ; PR-OD-1017-20002: AR). Royal Society (Dorothy Hodgkin Fellowship: RL; RP\EA\180004: PK). UK DHSC/UK Aid/NIHR (PR-OD-1017-20001: HPG). UK MRC (MC_PC_19065 - Covid 19: Understanding the dynamics and drivers of the COVID-19 epidemic using real-time outbreak analytics: AG, NGD, RME, TJ, YL; MR/P014658/1: GMK). Authors of this research receive funding from UK Public Health Rapid Support Team funded by the United Kingdom Department of Health and Social Care (TJ). Wellcome Trust (206471/Z/17/Z: OJB; 210758/Z/18/Z: JDM, KS, NIB, SA, SFunk, SRM). No funding (AMF, AS, CJVA, DCT, JW, KEA, YWDC).

Supplementary references

1 University of Liverpool. Liverpool Covid-SMART Pilot - Coronavirus (COVID-19). https://www.liverpool.ac.uk/coronavirus/research-and-analysis/covid-smart-pilot/ (accessed Dec 24, 2020).
2 Alemany A, Baro B, Ouchi D, *et al.* Analytical and Clinical Performance of the Panbio COVID-19 Antigen-Detecting Rapid Diagnostic Test. Infectious Diseases (except HIV/AIDS), 2020 DOI:10.1101/2020.10.30.20223198.
3 Ra SH, Lim JS, Kim G, Kim MJ, Jung J, Kim S-H. Upper respiratory viral load in asymptomatic individuals and mildly symptomatic patients with SARS-CoV-2 infection. *Thorax* 2021; **76**: 61–3.
4 Long Q-X, Tang X-J, Shi Q-L, *et al.* Clinical and immunological assessment of asymptomatic SARS-CoV-2 infections. *Nat Med* 2020; **26**: 1200–4.
5 Lee S, Kim T, Lee E, *et al.* Clinical Course and Molecular Viral Shedding Among Asymptomatic and Symptomatic Patients With SARS-CoV-2 Infection in a Community Treatment Center in the Republic of Korea. *JAMA Intern Med* 2020; **180**: 1447.
6 Zhou R, Li F, Chen F, *et al.* Viral dynamics in asymptomatic patients with COVID-19. *Int J Infect Dis* 2020; **96**: 288–90.
7 Pilarowski G, Marquez C, Rubio L, *et al.* Field performance and public health response using the BinaxNOW TM Rapid SARS-CoV-2 antigen detection assay during community-based testing. *Clin Infect Dis* 2020; : ciaa1890.
8 Pray IW, Ford L, Cole D, *et al.* Performance of an Antigen-Based Test for Asymptomatic and Symptomatic SARS-CoV-2 Testing at Two University Campuses — Wisconsin, September–October 2020. *MMWR Morb Mortal Wkly Rep* 2021; **69**: 1642–7.
